# Cross-Talks Between Macro- and Micronutrient Uptake and Signaling in Plants

**DOI:** 10.3389/fpls.2021.663477

**Published:** 2021-10-15

**Authors:** Xiaoning Fan, Xiaoqin Zhou, Hui Chen, Ming Tang, Xianan Xie

**Affiliations:** State Key Laboratory of Conservation and Utilization of Subtropical Agro-Bioresources, Guangdong Laboratory for Lingnan Modern Agriculture, Guangdong Key Laboratory for Innovative Development and Utilization of Forest Plant Germplasm, College of Forestry and Landscape Architecture, South China Agricultural University, Guangzhou, China

**Keywords:** nitrogen, phosphorus, sulfur, zinc, iron, nutrition cross-talks, nutrient homeostasis

## Abstract

In nature, land plants as sessile organisms are faced with multiple nutrient stresses that often occur simultaneously in soil. Nitrogen (N), phosphorus (P), sulfur (S), zinc (Zn), and iron (Fe) are five of the essential nutrients that affect plant growth and health. Although these minerals are relatively inaccessible to plants due to their low solubility and relative immobilization, plants have adopted coping mechanisms for survival under multiple nutrient stress conditions. The double interactions between N, Pi, S, Zn, and Fe have long been recognized in plants at the physiological level. However, the molecular mechanisms and signaling pathways underlying these cross-talks in plants remain poorly understood. This review preliminarily examined recent progress and current knowledge of the biochemical and physiological interactions between macro- and micro-mineral nutrients in plants and aimed to focus on the cross-talks between N, Pi, S, Zn, and Fe uptake and homeostasis in plants. More importantly, we further reviewed current studies on the molecular mechanisms underlying the cross-talks between N, Pi, S, Zn, and Fe homeostasis to better understand how these nutrient interactions affect the mineral uptake and signaling in plants. This review serves as a basis for further studies on multiple nutrient stress signaling in plants. Overall, the development of an integrative study of multiple nutrient signaling cross-talks in plants will be of important biological significance and crucial to sustainable agriculture.

## Introduction

In natural ecosystems, terrestrial vascular plants as sessile organisms are faced with highly variable environmental conditions and the consequent stresses associated with varying environmental signals, including soil nutrient-deficiency signals, which affect growth and development negatively (Bouain et al., 2019). Crop species in agricultural soils are subjected to various nutrient stresses during their lifecycle, such as lower availability of essential mineral elements, including nitrogen (N), phosphorus (P), sulfur (S), zinc (Zn), and iron (Fe). Thus, plants have evolved highly sophisticated mechanisms to coregulate these stresses in order to maintain homeostasis (Saenchai et al., 2016; Bouain et al., 2019; Xie et al., 2019).

Higher plants require at least 17 essential mineral elements for survival and development, including macro- and microelements (Marschner, 1995). In addition, the beneficial elements, such as selenium (Se) and silicon (Si), are important for optimal crop growth and production and are beneficial trace elements in human health (Meharg and Meharg, 2015; Zhou et al., 2020). A deficiency or excess of any of the mineral elements can result in physiological and metabolic disorders in plants, and adversely affect plant growth (White and Brown, 2010). However, nutrient availability is largely constrained by soil physicochemical properties (Alam et al., 1999; Kim et al., 2016); hence, plants have developed several mechanisms to cope with the changes, ranging from deficiency to excess (Maathuis, 2009; Krouk et al., 2011; Gruber et al., 2013).

In the last decades, the impact of nutrient deficiencies on crop growth and production has become a major concern, and the adverse effects threaten food safety and quality (Abelson, 1999; Neset and Cordell, 2012; Shahzad et al., 2014). To meet the global demand for food and agricultural raw materials, farmers rely heavily on the use of fertilizers to improve crop yield. However, long-term use of fertilizer is associated with negative ecological impacts, such as soil compaction and acidification, water loss, and soil erosion. The problems associated with the long-term use of fertilizers can be reduced by the use of nutrient-efficient crop varieties and previously uncultivated lands that are nutrient-rich for crop production. Therefore, for sustainable agriculture and reduced use of fertilizer, breeders and molecular scientists should focus on developing mineral-efficient crop varieties. However, to achieve this goal, an in-depth understanding of the response of plants to soil nutrient deficiency and the associated signaling pathways is necessary (Briat et al., 2015; Bouain et al., 2019).

Previous studies focused mainly on understanding the mechanisms and signaling pathways involved in maintaining homeostasis during a single nutrient deficiency in model plants, such as *Arabidopsis* and rice, without considering other nutrients or multiple nutrient-deficiency scenarios. However, the results of these studies have contributed to the knowledge of genes involved in maintaining mineral nutrient homeostasis during nutrient deficiency. Using molecular biology, genetics, and omics approaches, several key genes that regulate N, phosphate (Pi), Zn, and Fe uptake and homeostasis in *Arabidopsis* and rice plants during mineral deficiency have been identified (Kobayashi and Nishizawa, 2012; Park et al., 2014; Bouain et al., 2018; Yang et al., 2018). Mineral nutrient cross-talks in plants have been a topic of interest in plant nutrition. Over 7 decades, physiological and molecular studies have revealed the existence of antagonistic or synergistic relationships between macro- and micronutrients (Reed, 1946; Cakmak and Marschner, 1986; Huang et al., 2000; Misson et al., 2005; Zheng et al., 2009; Bouain et al., 2014a,b; Varala et al., 2018; Medici et al., 2019; Chaiwong et al., 2020). Recent findings on stresses associated with nutrient deficiency have indicated that plant growth response is remarkably affected by the complex cross-talks between N, Pi, Zn, and Fe uptake and homeostasis in plants. Pi has been reported to interact with Zn and Fe in plants (Zheng et al., 2009; Bouain et al., 2014a; Briat et al., 2015). However, studies on how plants integrate multiple nutrient signals into developmental programs, as well as the molecular processes underlying these complex cross-talks, are limited. Very recent investigation has suggested that variations in nutrient availability elicit the unique signatures of substantial transcriptional reprogramming, and gene co-expression analysis further suggests that master transcriptional regulators, which are the PIF4, HY5, and NF-Y responsible for light signaling (Siefers et al., 2009; Chen et al., 2016; Paik et al., 2017), coordinate plant growth and nutrient utilization in response to nutrient stresses (Brumbarova and Ivanov, 2019). Therefore, there is a need for integrative studies on the cross-talks between N, Pi, Zn, and/or Fe nutrient uptake and signaling in plants. The understanding of these cross-talks could be crucial to developing nutrient-efficient crops, with improved crop yield and quality.

In this review, we examined recent studies on the chemical and biochemical interactions between macro- and micro-elements in plants, physiological interactions among these mineral nutrients in plants, and focused on the physiological and molecular cross-talks between N, Pi, S, Zn, and Fe homeostasis to better understand how these nutrient interactions affect the mineral uptake and signaling in plants.

## Chemical and Biochemical Interactions Between Macro- and Micro-Elements in Plants

The physiological-biochemical connections have been built up between Pi and Fe in plants (Hirsch et al., 2006; Ward et al., 2008). First, the Pi-Fe complex forms the precipitates in rhizosphere soils, reducing the availability of the P and Fe elements for plants. As a result, the iron uptake system is activated in roots under Fe-limited and Pi-sufficient conditions (Ward et al., 2008; Briat et al., 2015). By contrast, Pi deficiency enhances Fe and aluminum (Al) accumulation in plants (Misson et al., 2005; Hirsch et al., 2006; Ward et al., 2008). On the other hand, plants have activated several forms of biochemical fitness to Pi deficiency. For example, plants produce the organic acids through aluminum-induced malate and citrate transporters (Delhaize et al., 2012) to increase the P solubility and make soil Pi available for root acquisition. Because organic acids have a high affinity for Calcium (Ca), Al, and Fe salts and can displace inorganic Pi from these precipitates (de Bang et al., 2021). Once Fe and Pi entered the roots, Fe can interact with Pi inside roots, resulting in a decreased Pi translocation to the aboveground tissues (Cumbus et al., 1977; Mathan and Amberger, 1977). A similar interaction has been observed in leaves, where high Pi promotes chlorosis, although Fe content is sufficient in leaves (Dekock et al., 1979). However, in seeds, Fe is stored in vacuoles in the form of inositol hexakisphosphate-Fe complex (Lanquar et al., 2005). These findings reveal that Pi is a chelator of Fe in plants. Therefore, the alterations in Pi homeostasis in plants significantly affect the availability of Fe, and these findings provide further evidence for the chemical interaction between Pi and Fe homeostasis in shoots and seeds.

From the biochemical viewpoint, in plants, Fe is considered to interact with S for the Fe sink formation of Fe-S clusters, which are necessary for many cellular enzymatic reactions during photosynthesis and respiration processes (Couturier et al., 2013; Briat et al., 2015; Przybyla-Toscano et al., 2021). It is of interest to study the potential role of the Fe-S cluster abundance in plants in response to different nutrient stresses in the future (Couturier et al., 2013; Forieri et al., 2013). On the other hand, sulfur deficiency interacts with the plant ionome, leading to a reduction in the photosynthetic carbon assimilation and metabolism processes (Courbet et al., 2019; Jobe et al., 2019; de Bang et al., 2021). The ferredoxin is an important component of the groups in Fe-S proteins and modulates electron transfer in the photosynthetic electron transport chain (Forieri et al., 2013; Zheng and Leustek, 2017). Therefore, S deficiency suppresses the photosynthetic efficiency and accelerates the chlorotic development in leaves (de Bang et al., 2021).

A large part of Fe and other micronutrients [such as Zn, manganese (Mn), and copper (Cu)] interactions could be explained by the competition of these ions by transceptors or sensors. Kobayashi et al. (2003) reported that tobacco plants subjected to combined deficiency of Fe, Zn, Mn, and Cu showed mitigated Fe-deficiency symptoms when compared with the individual Fe deficiency. Accordingly, the authors proposed that plant Fe sensors detect the cellular concentration ratio between Fe and other micronutrients rather than sole Fe concentration (Kobayashi et al., 2012, 2013; Kobayashi and Nishizawa, 2014).

However, a part of some other macro- and micro-elements interactions could be explained by competition of the metal ions by chelators or transporters. Ca modulates the leaf cell-specific phosphorus allocation. For example, Ca enhances the allocation of phosphorus to palisade mesophyll cells in Proteaceae under high P conditions (Hayes et al., 2019). In addition, cadmium (Cd) affects the translocation of some metals in either a Fe-like or Ca-like way in poplar plants and inhibits the transport of Ca-like metals competitively. The reduced translocation of chelator-dependent metals implicates Cd-related disturbances in the gene expression of xylem transporters or chelators (Solti et al., 2011). Furthermore, competition between uptake of ammonium and potassium exists in barley and *Arabidopsis* roots. It was shown that plant K^+^ transporters and channels could transport ${\text{NH}}_{4}^{+}$. Consequently, ${\text{NH}}_{4}^{+}$ uptake through the K^+^ transporters may contribute to ${\text{NH}}_{4}^{+}$ excess in the cytoplasm under K^+^ limitation; conversely, K^+^ application can alleviate the cellular ${\text{NH}}_{4}^{+}$ toxicity (ten Hoopen et al., 2010). From the chemical viewpoint, it has been shown that the chemical interactions that generate plant mineral nutrient dynamics can result from the chemical similarities or analogy between elements (Baxter, 2009; Courbet et al., 2019). It has been found the results of chemical analogy for elements-derived ions at the transporter level. For example, the chemical analogs of S (Se) and Ca (Sr) share the same transporter to take up these elements. Similarly, another example has been shown that phosphate transporters can take up phosphate as well as arsenate, resulting in the interaction between P and As (arsenic) in plants.

Overall, it is generally acknowledged that these macro- and micro-elements chemically or biochemically interact at various tissues throughout the life cycle of plants. However, the underlying molecular mechanisms regulating the integrated homeostasis of these elements remain to be deciphered.

## The Functional Ionome of Plants: The Complex Cross-Talks Between Macro- and Micronutrients

**Figure 1:**
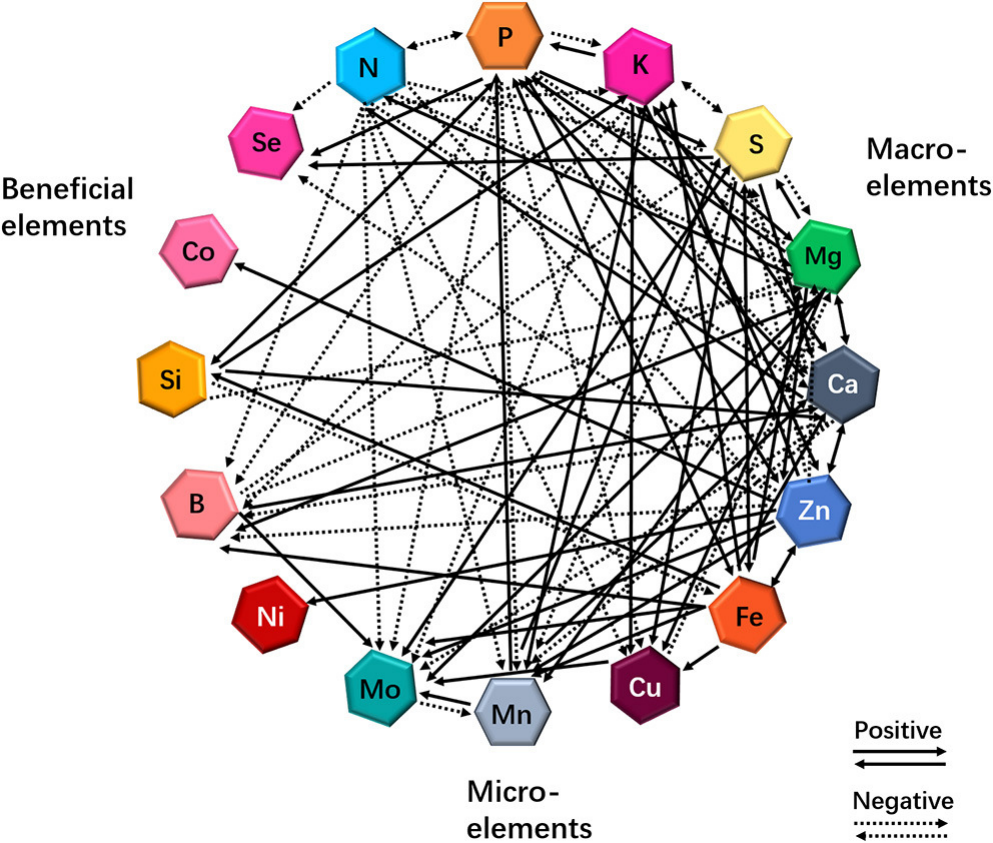
Cross-talks between macro- and micro-elements or beneficial elements in plants in response to individual mineral deficiency. Interactions resulting from single-element deficiency (any one of 16 elements) lead to enhanced (solid lines) or decreased (dashed lines) uptake of other minerals. Updated from the previous publications (Bokor et al., 2015; Rai et al., 2015; Maillard et al., 2016; Courbet et al., 2019; Chaiwong et al., 2020; Zhou et al., 2020; Ji et al., in press).

More than a decade ago, the “ionome” was defined as “the mineral nutrient and trace-element composition of a living organism” (Salt et al., 2008). Recently, the concept of functional ionome has been proposed by some scientists (White et al., 2017), and the functional ionome contains all the mineral elements, which are essential for the growth and development of living organisms. These elements can be classified into the macronutrients N, P, potassium (K), S, magnesium (Mg), and Ca, and into the micronutrients Zn, Fe, Cu, Mn, molybdenum (Mo), nickel (Ni), and boron (B) (White and Brown, 2010; Figure 1). Nevertheless, several earlier studies have revealed that three additional elements, including Si, cobalt (Co), and Se, are also essential for optimal plant growth (Epstein, 1999; White et al., 2004; Van Hoewyk et al., 2008; Tan et al., 2010; Figure 1).

It is well-known that the mineral element composition of a plant is highly regulated at different hierarchic levels (Lešková et al., 2017; Courbet et al., 2019). These sophisticated regulations allow plants to optimize ion uptake and protect plants from highly reactive ions. Andresen et al. (2018) reviewed such complex regulation of trace metal metabolism in plants. However, the multiple cross-talks between macro- and micronutrients have been uncovered in several local approaches; therefore, it is necessary for investigating the dynamics of plant ionome in a global way. A complete understanding of ionome homeostasis requires a thorough investigation of the dynamics of the nutrient networks in plants. Although the ionome homeostasis is very poorly understood, several recent studies have shown the diagram of the ionomic networks and multiple cross-talks of the ionome in plant species (Baxter, 2009; Kellermeier et al., 2014; Brumbarova and Ivanov, 2019; Courbet et al., 2019).

Nutrient deficiencies are able to modify the functional ionome of plant tissues (Maillard et al., 2016; Courbet et al., 2019). Maillard et al. (2016) identified 18 different interactions in rapeseed plants (*Brassica napus* L.) under mineral nutrient deficiency at the uptake level. Particularly, Mo uptake was significantly increased in plants under S, Fe, Zn, Cu, Mn, or B deficiency (Figure 1), and the authors proposed that this result could be the consequence of the direct and indirect disturbances of Mo and S metabolisms, resulting in the enhancement of Mo and ${\text{SO}}_{4}^{2-}$ transporters, respectively. On the other hand, S availability can modify the functional ionome of plants. Although many studies have focused on the effects of S deficiency on mineral nutrients in plants at the transcriptomic or metabolic levels in different plants in the past 2 decades (Hirai et al., 2003; Ciaffi et al., 2013; Wipf et al., 2014; Forieri et al., 2017), several reports have started to consider the consequences of S deficiency on the leaf ionomic composition of *B. napus* plants (Maillard et al., 2015, 2016). When the plants are exposed to S deficiency, several positive and negative interactions between S and other mineral nutrients have been presented, as illustrated in Figure 1, which is compiled from different studies (Abdallah et al., 2010; Bokor et al., 2015; Rai et al., 2015; Maillard et al., 2016; Courbet et al., 2019; Chaiwong et al., 2020; Zhou et al., 2020; Ji et al., in press). Moreover, Si and Zn cross talks influence the functional ionome in maize (*Zea mays*). Supplying maize plants with Si and/or Zn significantly decreased the concentration of Pi, K, Mg, Ca, Mn, Ni, and Co in the roots but increased the concentration of Se (Bokor et al., 2015; see Figure 1). Conversely, the positive effects of Si and Fe interaction on the growth and production of plants have been reported in vegetables and crops (Gonzalo et al., 2013; Pavlovic et al., 2013; Dragama et al., 2019). Therefore, the dynamics in the functional ionome of plants subjected to individual mineral deficiency indicate that the complexity and the diversity of interactions occur between single and other mineral nutrients in plants.

Summarily, these ionomic analyses further support the occurrence of complex cross-talks between mineral nutrients in plants, indicating that, under certain deprivations, nutrient cross-talks stimulate or reduce the accumulation of other mineral nutrients, which modifies the ionomic composition of plant tissues (see Figure 1). In addition, some studies have examined the physiological and genetic interactions between elements in plant nutrition using combined ionomics and genome-wide association study (GWAS) approaches (Baxter et al., 2008; Yang et al., 2018). Applying low Pi in *Arabidopsis thaliana* growth culture medium affected the concentrations of other nutrients, with a considerable increase in the concentrations of Zn, Fe, and S, and a decrease in the concentrations of copper and cobalt in the experimental plants (Baxter et al., 2008). Owing to the rapid development of multiple omics and GWAS approaches and systems biology (Rouached and Rhee, 2017), progress has been made in understanding the molecular mechanisms underlying the physiological and genetic processes resulting from multiple nutrient signals.

## Interaction Between Pi, S, and Fe Nutrient Homeostasis in Plants

**Figure 2:**
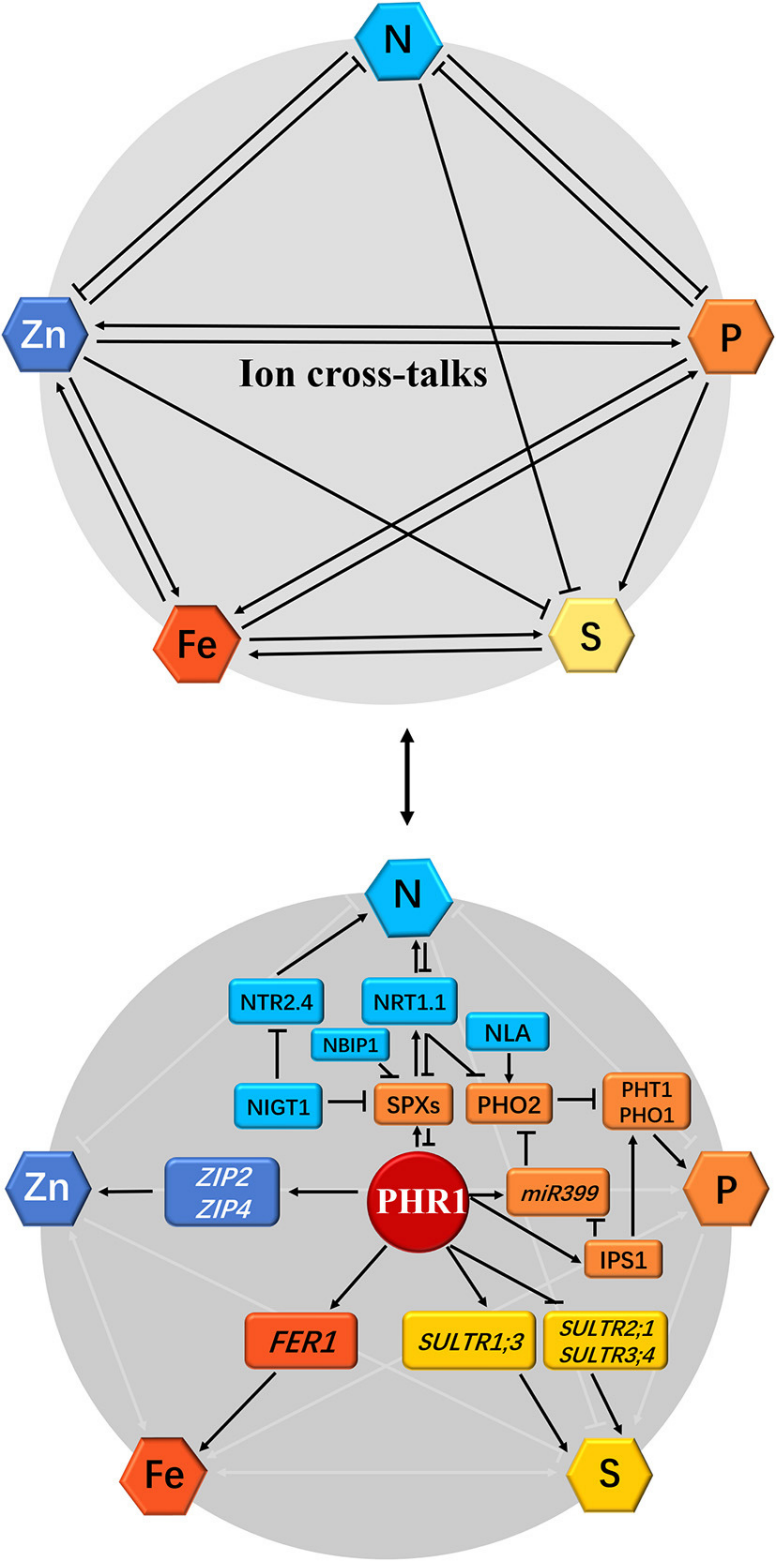
Schematic representation of the effect of individual mineral deficiency on N, P, S, Zn, and Fe homeostasis and gene regulatory networks in plants. The interactions between nitrogen (N), phosphorus (P), sulfur (S), zinc (Zn), and iron (Fe) nutrient homeostasis are shown at the physiological level by arrows or flat-ended lines (the above panel). The single-direction arrows and flat-ended lines indicate the antagonistic and synergistic interactions, respectively. For the molecular bases of cross-talks among these five mineral nutrients, the phosphate (Pi)-starvation-induced transcription factor PHR1 acts as a potential integrator of N, P, S, Zn, and Fe nutrient signals in plants (the below panel): (1) During Pi deficiency, the activation of PHR1 induces Pi transporters PHT1 and PHO1 as well as Pi-starvation response genes SPXs, miR399, and IPS1 through both PHR1-P1BS and PHR1-miR399-PHO2 pathways, and represses PHO2, which interacts with the E3 ligase NLA to degrade Pi transporters, whereas PHR1 is inhibited by SPXs under Pi sufficiency. (2) N supply promotes PHR1 activity, however, SPXs expression is directly reduced under such a scenario by NIGT1 (Hu et al., 2019 reported that rice OsSPX4 is degraded in response to N supply by the interaction of OsNRT1.1B and OsNBIP1). Simultaneously, PHO2 expression is repressed in response to high N by NIGT1 and NRT1.1. Accordingly, N starvation leads to the decreased Pi uptake in plants due to the inhibition of PHR1 and accumulation of SPXs and PHO2. (3) PHR1 also serves as a transcriptional regulator of the sulfate transporters. (4) Plant Fe homeostasis is also dependent on the PHR1, which directly upregulates the Fe storage protein FER1 expression. (5) Zn transport and homeostasis are also involved in a PHR1-dependent manner; under Pi starvation, PHR1 positively regulates the expression of zinc transporter genes *ZIP2* and *ZIP4* through binding to the P1BS *cis*-elements in these gene promoters (Briat et al., 2015; Kumar et al., 2021; see following Figure 3A). The arrows and flat-ended lines indicate the positive and negative influences, respectively.

Plant responses to Pi and S signals have been studied in *Arabidopsis*. Rouached et al. (2011) reported an increase in root ${\text{SO}}_{4}^{2-}$ concentration and a decrease in shoot ${\text{SO}}_{4}^{2-}$ concentration during Pi deficiency, indicative of the regulation of the ${\text{SO}}_{4}^{2-}$ transport process in Pi-deficient plants. Additionally, the mechanism of ${\text{SO}}_{4}^{2-}$ transport in the Pi-deficient plants was regulated by the PHOSPHATE DEFICIENCY RESPONSE 1 (PHR1) factor, which is an MYB protein that regulates the response of plants to Pi deficiency. PHR1 regulated the expression of the ${\text{SO}}_{4}^{2-}$ transporter-encoding gene, *Sultr1;3*, and inhibited the expression of *Sultr2;1* and *Sultr3;4* during Pi deficiency (Figure 2). Taken together, these findings suggest the presence of a complex process in the co-regulation of Pi and ${\text{SO}}_{4}^{2-}$ homeostasis in plants.

Acquisition of S, K, and Fe by roots is of crucial importance for plant growth and yield, and recent studies have revealed that the uptake systems for S and Fe nutrients are coordinated in plants (Astolfi et al., 2010; Zuchi et al., 2015). Moreover, Forieri et al. (2017) demonstrated that the plant is differentially co-regulated upon long-term Fe, S, and K deficiency through the systematic analyses of metabolism and the transcriptome in roots. The authors found the specific co-regulation between the S and Fe metabolic pathways in roots upon S or Fe deficiency. Interestingly, Fe deficiency regulated a distinct subset of the S assimilation genes that were not controlled by S deficiency itself, suggesting the presence of two independent signaling pathways in this network. In particular, the cross-talk between S and Fe pathways in roots existed an opposing regulation component that was upregulated during S deficiency and downregulated during Fe deficiency or *vice versa*. These opposing expression genes were involved in the S and Fe assimilation pathways, for example, the opposing regulation upon Fe and S deficiency was found for key components of the sulfate-starvation response (*SULTR1;1* and *APR* genes) and the Fe uptake systems (*FRO2* and *IRT1*) and NA synthesis (*NAS4*) (Forieri et al., 2017), demonstrating the presence of specific signaling cascades for the cross-regulation of the S and Fe routes. It was also found that S deficiency depressed the Fe assimilation pathway, including the well-known Fe transcription factors-encoding genes *FIT, PYE, BTS, bHLH38, bHLH100*, and *bHLH101*, and Fe import gene *IRT1*. In contrast, the simultaneous S and Fe deficiencies alleviated the negative effects of single S deficiency on transcription of the Fe assimilation pathway (such as *FRO2, FIT, PYE, bHLH39, bHLH100, NAS1*, and *NAS4*) and *vice versa*, and led to the typical induction of both S and Fe nutrient uptake systems. This demonstrated that specific nutrient-deficiency response signaling (such as induction of *FIT* in double S and Fe deficiencies) can control this cross-regulation. On the other hand, fewer S- or Fe-deficiency-responsive genes were co-regulated in response to the K deficiency, while the genes involved in the S and Fe assimilation pathways were almost unaffected by low K application (Forieri et al., 2017). Therefore, the response to K deficiency shown did not participate in the cross-talk between S and Fe nutrients. In addition, a significant decrease of total sulfate in roots in response to K deficiency may be explained by the downregulation of important key components of the sulfate assimilation pathway upon K deficiency (e.g., *SULTR1;1, APK4*, and *SOT7*), providing evidence for the specificity of the K and S cross-talk, whereas the K-deficiency response resulted in a strong increase in the content of reduced S-containing metabolites (Forieri et al., 2017), demonstrating a shift of the oxidized sulfate to the reduced state. This can explain the unaltered total S concentration in roots during K deficiency.

## Interaction Between N and Pi Nutrient Homeostasis in Plants

N and P are two essential macronutrients necessary for plant growth and productivity, and a deficiency in any of the two nutrients may negatively affect plant growth and yield. The earlier reports revealed a mutual interaction between N and P nutrition in plants under diverse ecosystems (Gniazdowska and Rychter, 2000; Elser et al., 2007). Recent studies have shown that *Arabidopsis* and crop species possess highly developed mechanisms for maintaining Pi and N homeostasis in response to Pi- and N-deficiency signaling (Lin et al., 2013; Park et al., 2014; Medici et al., 2019). It was reported that interaction between N and P signaling in plants was mediated by nitrogen limitation adaptation (NLA), a RING-type E3 ubiquitin ligase, which is involved in N-dependent P accumulation in shoots by promoting the degradation of PHT1 proteins with the help of PHO2 (Lin et al., 2013; Park et al., 2014; also see Figure 2). Medici et al. (2019) reported that N signaling regulates phosphate-deficiency response (PSR) in rice (*Oryza sativa*) through three molecular integrators (PHR1, PHO2, and NRT1.1). N signaling regulates N-P cross-talk in rice by regulating the accumulation and turnover of PHR1 (Figure 2). Furthermore, phosphate2 (PHO2), an E2 ubiquitin conjugase, functions as an important integrator of N signaling in PSR. Moreover, PHO2 is also involved in regulating the activities of rice nitrate transceptor, NRT1.1, suggesting that NRT1.1 is a component of N-P-signaling cross-talk in plants. The mechanism underlying PSR activities through nitrogen signaling is conserved in *O. sativa* and wheat (*Triticum aestivum*) species (Medici et al., 2019). Hu et al. (2019) found that the rice nitrate transporter OsNRT1.1B interacts with OsSPX4 to activate the OsPHR2 activity in response to nitrate supply. Subsequently, OsSPX4 is ubiquitinated by the NRT1.1B interacting protein 1 (NBIP1) and degraded through the 26S proteasome pathway. Therefore, high N induces the PHR1/2 activity to promote Pi starvation responses and enhance Pi uptake in plants. At the transcriptional level, high N availability induces the expression of genes-encoding PHR proteins, such as AtPHL1 and OsPHR3 (Sun et al., 2018; Varala et al., 2018). Furthermore, the nitrate-inducible GARP-type transcriptional repressor 1 (NIGT1) proteins bind to and repress the promoters of *SPX* genes in *Arabidopsis* to modulate the PHR activity, *PHT1;1* expression, and Pi acquisition (Ueda et al., 2020; also see Figure 2). Finally, these findings are summarized as follows: (i) N application increases the PHR1 protein stability in plants (Medici et al., 2019; Kumar et al., 2021); (ii) NRT1.1 interacts with SPX to degrade the SPX protein during nitrate supply (Hu et al., 2019; Kumar et al., 2021); and (iii) the nitrate–NIGT1–SPX–PHR1–PHT1 signaling is the key regulatory pathway that mediates Pi uptake in response to N availability (Ueda et al., in press). These molecular actors that integrate N and P signals into crops may have implications in biotechnology and agricultural practices.

## Interaction Between N and Zn Nutrient Homeostasis in Plants

Apart from N and P, Zn is one of the most yield-limiting nutrients in crop species, and N is a critical player in Zn uptake and translocation in plants (Erenoglu et al., 2011). Recent studies on N-Zn cross-talk have confirmed that N application improves Zn uptake and homeostasis in rice and wheat plants under Zn deficiency (Kutman et al., 2011; Ali et al., 2014), and that N and Zn applications increase grain crude protein as well as N and Zn concentrations significantly (Nie et al., 2018). Moreover, an earlier study found that low Zn supply significantly reduced the nitrate uptake capacity of cotton, sunflower, and buckwheat (Cakmak and Marschner, 1990). A more recent report has shown that moderately high Zn supply increased the root-to-shoot translocation of N into the leaves and brown rice and enhanced the rice yield; synergistically, N application significantly promoted the root-to-shoot translocation of Zn into the leaves and brown rice (Ji et al., in press). These findings reveal that N and Zn act synergistically on root-to-shoot translocation and preferential distribution in grains of crop species (Figure 2). However, the molecular mechanism underlying the cross-talk between N and Zn uptake and homeostasis in plants is poorly understood. Therefore, an understanding of the molecular mechanisms underlying plant N and Zn homeostasis will contribute to improving plant Zn nutrition. Recently, transcriptome analyses of nitrate and Zn deficiency in plants have provided insights into the mechanisms regulating the cross-talk between N and Zn homeostasis (Azevedo et al., 2016; Varala et al., 2018). The transcriptomic data revealed that the expression of numerous N transport and homeostasis-related genes were upregulated in the shoots of *Arabidopsis* plant during Zn deficiency. The *Arabidopsis* nitrate transporter 2.4 gene (*NRT2.4*, AT5G60770) (Wang et al., 2018), which is a marker gene in N-deficiency response, was highly upregulated by Zn deficiency. Furthermore, these expression patterns rely on two critical bZIP transcription factors, bZIP19 and bZIP23, which regulate the adaptation of plants to Zn deficiency (Assuncao et al., 2010). Moreover, the two bZIP transcription factors function as transcriptional suppressors in the negative regulation of ammonium transporters-encoding genes, *AMT1.1* (AT4G13510) and *AMT2.1* (AT2G38290), during Zn deficiency (Wang et al., 2018). Contrarily, the expression of *AMT1.1* and *AMT2.1* was upregulated in *bzip19* × *bzip23* double mutants compared with that in the wild type (Azevedo et al., 2016). These findings indicate that the molecular mechanism underlying N deficiency was initiated in Zn-deficient plants. N deficiency reduced the expression of the Zn-transport-related gene, encoding ferric reductase defective3 (FRD3) MATE transporter (AT3G08040), which participates in the process of Zn loading into the xylem (Pineau et al., 2012). The physiological and transcriptional results presented here provide new insights into the complexity of N and Zn interactions in plants. The molecular linkers identified in the reviewed studies may contribute to the understanding of the cross-talk between N and Zn signaling.

## Cross-Talk Between Pi, Zn, And Fe Homeostasis in Plants

Cross-talk between macro- and micronutrients and their influence on ion accumulation in plants have been partially studied. Hence, this review further focused on the interaction between Pi, Zn, and Fe homeostasis at the physiological and molecular levels. Recent studies have shown that the interactions of nutrients are a common process in plants (Briat et al., 2015; Rouached and Rhee, 2017; Bouain et al., 2019). Pi-deficiency results in Zn over-accumulation in the shoots of plants, and *vice versa* (Bouain et al., 2014a; Khan et al., 2014; Ova et al., 2015), indicating the antagonistic effect of Pi and Zn nutrition in plants. Furthermore, there is evidence of similar physiological cross-talk between Pi and Fe (Zheng et al., 2009) and between Fe and Zn (Haydon et al., 2012). Moreover, a few studies have examined the complex tripartite interactions between Pi, Zn, and Fe in plants (Briat et al., 2015; Rai et al., 2015; Saenchai et al., 2016). Although interactions between Pi, Zn, and Fe have been reported in plants, the molecular mechanisms underlying their actions are poorly understood. However, transcriptomic and genetic analyses of signaling cross-talks between Pi, Zn, and/or Fe nutrients in different plants could improve the understanding of the mechanisms underlying their interaction and activities (Hammond et al., 2003; Wu et al., 2003; Misson et al., 2005; van de Mortel et al., 2006; Zheng et al., 2009; Bustos et al., 2010; Thibaud et al., 2010; Rouached et al., 2011; Pineau et al., 2012; Khan et al., 2014; Moran et al., 2014; Li and Lan, 2015; Rai et al., 2015; Saenchai et al., 2016; Kisko et al., 2018; Bouain et al., 2019). These studies examined above have shown that signaling cross-talks between Pi, Zn, and/or Fe nutrients in plants are well-regulated in response to combined nutrient stress in the surrounding soils. The understanding of the cross-talks between Pi, Zn, and/or Fe nutrients in plants will be crucial in developing nutrient-efficient crop varieties.

## Interactions Between Pi and Zn Nutrients in Plants

### Pi Availability Affects Zn Uptake in Plants

Plant growth and development depend on soil Pi content; however, in agroecosystems, plants are often faced with Pi deficiency. In response to Pi deficiency, plants have evolved tightly controlled mechanisms to maintain Pi homeostasis, including the acquisition of Pi from the soil, storage and remobilization, and utilization of Pi to optimize metabolic processes (Poirier and Bucher, 2002). Nutrients interact with each other in plants, as the supply of one nutrient may affect the uptake of another nutrient. In wheat and maize, Pi and Zn have been shown to have antagonistic reaction, as an increase in Zn concentration results in a decrease in Pi concentration, and *vice versa* (Robson and Pitman, 1983; Verma and Minhas, 1987). Studies on *A. thaliana* showed a negative correlation between Pi and Zn concentration (Misson et al., 2005; Khan et al., 2014). Although this interaction is known as Pi-induced zinc deficiency, the relationship is complex (Marschner, 2012). Misson et al. (2005) found that long-term exposure of *A. thaliana* to phosphorus deficiency resulted in a higher concentration of Zn in the shoots and reduced biomass. Although higher levels of Pi increase grain yield in soil-grown plants, the Zn content of shoots decreases with increasing concentrations of Pi. However, if the available Zn is low, this effect can exacerbate Zn deficiency, as low Pi concentrations will initiate Zn uptake. Under both controlled and field conditions, higher soil Pi was associated with lower Zn concentration in wheat tissue (Thompson, 1990; Zhu et al., 2001; Zhang et al., 2012). High Zn concentration did not significantly affect the yield of wheat grown under low Pi condition, indicating that Pi was the yield-limiting nutrient (Ova et al., 2015). Furthermore, Zn deficiency and high Pi concentration result in Pi toxicity in plants. Pi-Zn interaction has also been identified in several biological systems (Marschner, 2012; Kisko et al., 2018). In *Zea mays*, high levels of Pi can immobilize Zn in roots and nodes by increasing the Zn-binding properties of the cell wall (Dwivedi et al., 1975; Youngdahl et al., 1977; Bouain et al., 2014a) reported a decrease in the Zn concentration of lettuce roots with an increase in Pi concentration. These data indicate that Pi nutrition may directly influence the Zn uptake mechanisms in plants. A mild Zn deficiency in Pi-deficient plants can increase the abundance of high-affinity Pi transporters without accumulating excess phosphorus in the plant (Huang et al., 2000).

### Zn Availability Affects Pi Uptake in Plants

Zinc is an essential micronutrient for all organisms and is involved in the growth and development of plants (Vallee and Auld, 1990; Andreini et al., 2007). Zn acts as a catalytic or structural cofactor in many enzymes and regulatory proteins (Maret, 2009). When grown in Zn-deficient soil, plants exhibit the enhancing Pi-uptake capacity. Studies have shown the higher Pi concentrations in the shoots of both dicotyledon and monocotyledon plant species in response to Zn deficiency (Welch et al., 1982; Cakmak and Marschner, 1986; Webb and Loneragan, 1988; Welch and Norvell, 1993). Moderate Pi levels increased the dry weight of Zn-deficient plants; however, at high-Pi levels, this effect was reversed, resulting in a decrease in shoot biomass. Contrarily, the biomass yield of plants was higher under moderate and high Zn concentrations (Ova et al., 2015). The effects of Zn deficiency in *A. thaliana* are exacerbated by an increase in Pi concentration in the shoots (Kisko et al., 2018). Several studies have shown that Zn deficiency positively affects root Pi uptake (Reed, 1946; Loneragan et al., 1982; Cakmak and Marschner, 1986; Huang et al., 2000; Khan et al., 2014). Zn deficiency significantly increased the Pi content of the shoots of *A. thaliana* wild-type and *pho2* mutant plants when compared with that in the *phr1* or *pho1* mutant (Bouain et al., 2014b). On the other hand, more recent study has shown that a high level of Zn decreased the rice-yield production and triggers P starvation by inhibiting root-to-shoot translocation and distribution of P into leaves by downregulating Pi transporter genes *OsPT2* and *OsPT8* in shoots of rice plants (Ding et al., 2021). By contrast, P supply decreased the Zn concentrations in rice plants by inhibiting the expression levels of the ZIP family Zn transport genes *OsZIPs* in roots (Ding et al., 2021). This study revealed the physiological and molecular mechanisms of a cross-talk between P and Zn in rice plants at the transport level and indicated that manipulating P fertilizer is an effective strategy to inhibit the toxic Zn uptake into plants when exposed to Zn excess in soil.

### Molecular Basis of Interaction Between Pi and Zn in Plants

**Figure 3:**
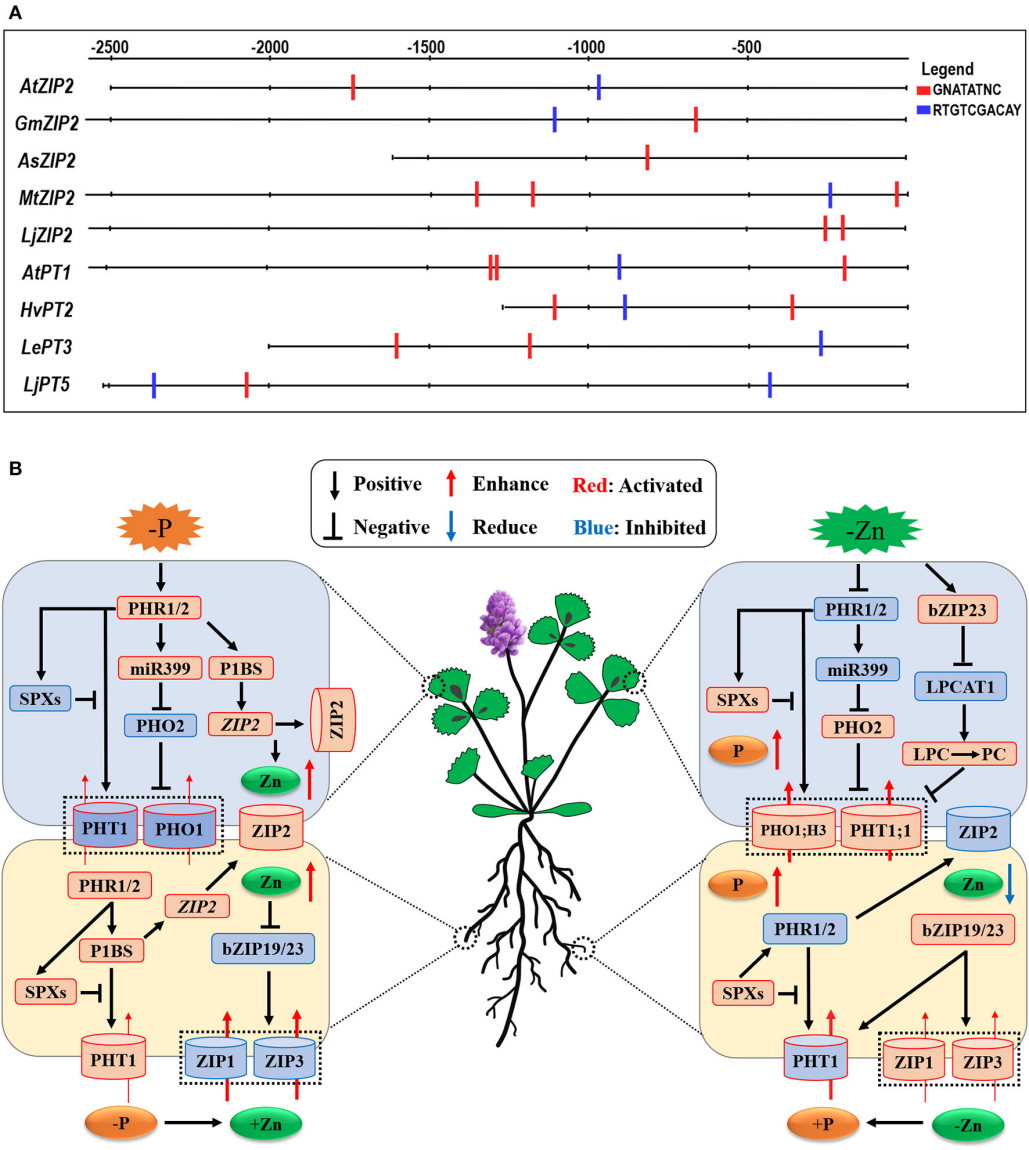
Schematic representation of the cross-talk between Pi and Zn nutrients and the potential linkers involved in the interaction between Pi and Zn nutrient homeostasis in plants. **(A)** the potential Pi- and Zn-deficient *cis*-regulatory elements present in the promoter regions of PHT1 family and ZIP family genes from plants. P1BS-motif (GNATATNC), which is the target site of PHR1 in plants, was screened in the promoter region of plant *PHT1* and *ZIP* genes by DNA-pattern-matching analysis (http://rsat.ulb. ac.be/rsat/). The GNATATNC motif is found in the promoter region of *AtZIP2* (in *Arabidopsis thaliana*), *GmZIP2* (in *Glycine max*), *AsZIP2* (in *Astragalus sinicus*), *MtZIP2* (in *Medicago truncatula*), and *LjZIP2* (in *Lotus japonicus*), while the promoter sequences of *AtPT1* (in *Arabidopsis thaliana*), *HvPT2* (in *Hordeum vulgare*), *LePT3* (in *Solanum lycopersicum*), and *LjPT5* (in *Lotus japonicus*) further contain the ZDRE motif (RTGTCGACAY), which is activated by the bZIP19/23 transcription factors during Zn deficiency. **(B)** Under Pi deficiency, the *PHT1* and *PHO1* genes are activated to be essential for Pi uptake and accumulation in plants through the Pi regulatory-signaling pathway (PHR1-miR399-PHO2); moreover, the ZIP2 subfamily genes, belonging to the plant ZIP gene family, are transcriptionally induced *via* the activated PHR1 transcription factor binding to the P1BS (GNATATNC) sequences found in the promoter regions of their genes (see **A**). On the other hand, Zn deficiency activates the bZIP19/23 transcription factors, and bZIP19/23-mediated Zn regulatory pathway induces the ZIP gene family transporters to promote Zn uptake and homeostasis. On the other hand, *LPCAT1* (*Lyso-PhosphatidylCholine AcylTransferase 1*), *PHT1;1*, and *PHO1;H3* are transcriptionally upregulated in a bZIP19/23-dependent manner during Zn deficiency. The black arrows and flat-ended lines refer to the positive and negative interactions, respectively. The red and blue arrows represent the increased and decreased element contents; accordingly, the red and blue modules indicate the activated and inhibited processes in plants.

Some studies have examined the interaction between Pi and Zn homeostasis in plants at the molecular level (Bouain et al., 2014a; Kisko et al., 2015). The expression of Pi uptake transporter genes is tightly controlled in roots in response to the P status of the plants (Franco-Zorrilla et al., 2004); however, this mechanism is lost under Zn deficiency. Very recently, Ueda et al. (in press) have proposed that Zn deficiency modulates the Pi uptake and Pi starvation response in plants. Under Pi deficiency, the transcription factors PHR1 is activated to accelerate the Pi starvation response in plants (Figure 3B, the left panel). The role of PHR1 in the coordination of Pi-Zn homeostasis has been demonstrated (Khan et al., 2014), while the expression of *PHT1* and *PHO1* genes is essential for Pi uptake and accumulation in plants through the Pi regulatory-signaling pathway (PHR1-miR399-PHO2) (Fujii et al., 2005; Aung et al., 2006; Bari et al., 2006; Chiou et al., 2006; Khan et al., 2014; Liu et al., 2014). In addition, the P-starvation-signaling PHR1-P1BS module in both roots and shoots leads to the Zn overaccumulation in plants as this module upregulates the ZIP2 subfamily genes belonging to the ZIP family (Briat et al., 2015; Kumar et al., 2021). Moreover, these authors proposed that plant *ZIP2* and *ZIP4* genes may be transcriptionally induced *via* the activated PHR1 transcription factor (Briat et al., 2015; Kumar et al., 2021). This proposition is consistent with our finding that at least one P1BS (GNATATNC) *cis*-element, which is directly bound by the PHR1, is present in the promoter regions of plant ZIP2 subfamily genes (see Figure 3A), implying that Zn transporter ZIP2 may regulate plant Zn homeostasis in a phosphorus-dependent manner. As a result, a high level of Zn in plants inhibits the root-to-shoot translocation and distribution of Pi into leaves through downregulating the PHT1 transporter genes in shoots (Ding et al., 2021).

On the other hand, a high level of Pi treatment results in repressed the PHR1/2-miR399-PHO2 and PHR1/2-P1BS modules in shoots and roots; consequently, Zn concentrations are largely decreased in both roots and shoots. This conclusion is also confirmed by some scientists who reported the decreased Zn-uptake ability in rice roots through the OsPHR2-dependent *ZIP* gene expression (Ding et al., 2021). Accordingly, Zn deficiency activates the bZIP19/23 transcription factors, which are the central controls of Zn-deficiency signaling and act as Zn sensors to regulate plant Zn availability (Lilay et al., 2021), and, subsequently, the bZIP19/23-mediated Zn regulatory pathway activates the ZIP gene family transporters to promote Zn uptake and homeostasis (Assuncao et al., 2010). However, under such a Zn-deficient scenario, the accumulation of Pi is also increased in leaves (Khan et al., 2014). In dicots, phosphate exporter PHO1, H3 engages in xylem loading of Pi and mediates Zn-deficiency-induced Pi accumulation in leaves, which is accompanied by an increase in the *PHT1;1* expression in shoots (Khan et al., 2014; see Figure 3B, the right panel), suggesting that these important genes play key roles in regulating Pi homeostasis and Zn-deficiency response in plants. Moreover, a recent investigation has revealed that a *Lyso-PhosphatidylCholine (PC)AcylTransferase 1*(*LPCAT1*) gene, which encodes the enzyme that converts Lyso-PhosphatidylCholine (LPC) to PhosphatidylCholine (PC), acts as the key determinant of leaf Pi accumulation under Zn deficiency (Kisko et al., 2018). These authors found the regulation of *LPCAT1* expression in a bZIP23 transcription factor (or zinc dependent) manner, for which they identified a novel bZIP23-binding motif upstream of the *LPCAT1* gene to repress its own expression. Under Zn-deficient conditions, loss of *LPCAT1* function enhances the contents of phospholipid LPC as well as the expression of the phosphate transporter PHT1;1, consequently, increases leaf Pi accumulation (Kisko et al., 2018). Based on the above, it is proposed that the LPC/PC ratio may affect the expression of *PHT1;1* in plant shoots. Because Drissner et al. (2007) reported that LPC as a signal elicits the arbuscular mycorrhiza-inducible phosphate transporter genes *StPT3/4* in roots of *Solanum tuberosum*, collectively, these new findings uncover that a Zn-regulated bZIP23-LPCAT1-(Lyso-PC/PC)-PHT1;1 cascade controls the shoot phosphate homeostasis (Figure 3B, the right panel).

## Interactions Between Pi and Fe Nutrients in Plants

### Pi Availability Affects Fe Uptake and Homeostasis in Plants

The uptake of elements by plants not only depends on the availability of elements in the soil solutions but also on the nutrient uptake capacity of the roots. Pi deficiency or excess can affect Fe homeostasis of plants (Liu et al., 2000; Zheng et al., 2009). Fe plays an important role in plant growth, development, and productivity. Although it is present in large quantities in soils, its absorption by plants is limited (Guerinot and Yi, 1994; Colombo et al., 2014). Fe can interact with phosphorus in the soil or growth medium, root surface, and in systems of plants (Ward et al., 2008; Zheng et al., 2009; Bournier et al., 2013; Rai et al., 2015). Higher concentrations of Fe have been observed in Pi-deficient plants, and this is attributed to the activities of Fe-responsive genes in response to Pi deficiency (Zheng et al., 2009); however, this phenomenon was not observed in high Pi medium (Wasaki et al., 2003; Misson et al., 2005; Hirsch et al., 2006). The absence of Fe in Pi-deficient medium promotes plant growth (Ward et al., 2008). In experimental conditions, the absence of Pi increased the Fe concentration of the shoots of seedlings; however, Fe concentration in the roots was unaffected, indicating that Pi and Fe have an antagonistic relationship (Zheng et al., 2009, Chaiwong et al., 2018). Therefore, regulating Pi homeostasis has a considerable effect on the availability of Fe (Bournier et al., 2013). Fe was present only in the vacuoles under high-Pi conditions, whereas several large grains of starch typical of Pi-starved plants were observed in most of the chloroplasts under low-Pi conditions (Hirsch et al., 2006).

### Fe Availability Affects Pi Uptake and Homeostasis in Plants

When both iron and phosphorus are absorbed by plants, the retention of Pi in roots increases and the transfer of Pi to the ground decreases in a concentration-dependent manner. Fe promoted Pi retention in the roots of apple plants and reduced Pi translocation to the shoots (Cumbus et al., 1977; Mathan and Amberger, 1977); By contrast, there was overaccumulation of Pi in both the roots and shoots of Fe-deficient apple plants. Pi and Fe interacted in an antagonistic manner to regulate the growth of rice shoots (Zheng et al., 2009). An antagonistic interaction between Fe and Pi contents was previously reported in crop species and apple trees (Zheng et al., 2009; Zanin et al., 2017; Valentinuzzi et al., 2019). The results of experiments on Pi- and Fe-treated *Arabidopsis* seedlings indicated that the availability of Fe affected the response of lateral roots to Pi deficiency (Rai et al., 2015). Recently, it has been suggested that iron-dependent callose deposition adjusts root meristem maintenance to Pi availability (Müller et al., 2015).

### Molecular Basis of Pi and Fe Interactions in Plants

Transcriptomic analysis of Pi-deficient plants showed that there was an increase in the expression of genes-regulating excess Fe and a reduction in the expression of genes-regulating iron deficiency (Misson et al., 2005; Müller et al., 2007; Thibaud et al., 2010). In this condition, the *AtFER1* gene, encoding the ferritin protein for Fe storage in *Arabidopsis*, was induced by Pi deficiency. Moreover, it was demonstrated that this *AtFER1* in response to Pi starvation was mediated by PHR1 and PHL1, which are two members of the MYB transcription factor family controlling Pi deficiency in plants, through their binding to the *cis*-regulatory element P1BS found in the promoter of *AtFER1*, regardless of Fe availability (Bournier et al., 2013). However, this Pi-deficient induction of *AtFER1* does not need the Fe-dependent *cis*-element IDRS found in its promoter (Bournier et al., 2013). On the other hand, the upregulation of *AtFER1* in response to excessive Fe is unchanged in single *phr1* (Bournier et al., 2013) or in double *phr1* × *phl1* (Bustos et al., 2010) mutant plants. These findings revealed that PHR1 and PHL1 are involved in Pi and Fe homeostasis interactions (Briat et al., 2015).

Genome-wide analysis in wild-type plants (Misson et al., 2005; Müller et al., 2007; Thibaud et al., 2010) and *phr1* × *phl1* double mutants (Bustos et al., 2010) showed that the expression of genes involved in iron homeostasis, such as *NAS3* and *YSL8*, was enhanced during Pi deficiency in wild-type plants, whereas they showed a decreased expression in the double-mutant plants. Furthermore, the genes in response to Fe deficiency, such as *FRO3, FOR6, IRT1, IRT2*, and *NAS1*, were inhibited by Pi deficiency in the wild-type plants, while they were out of control in the *phr1* × *phl1* double mutants (Bustos et al., 2010). These findings confirm that PHR1 and PHL1 act as integrators of Pi and Fe homeostasis interactions in plants.

**Figure 4:**
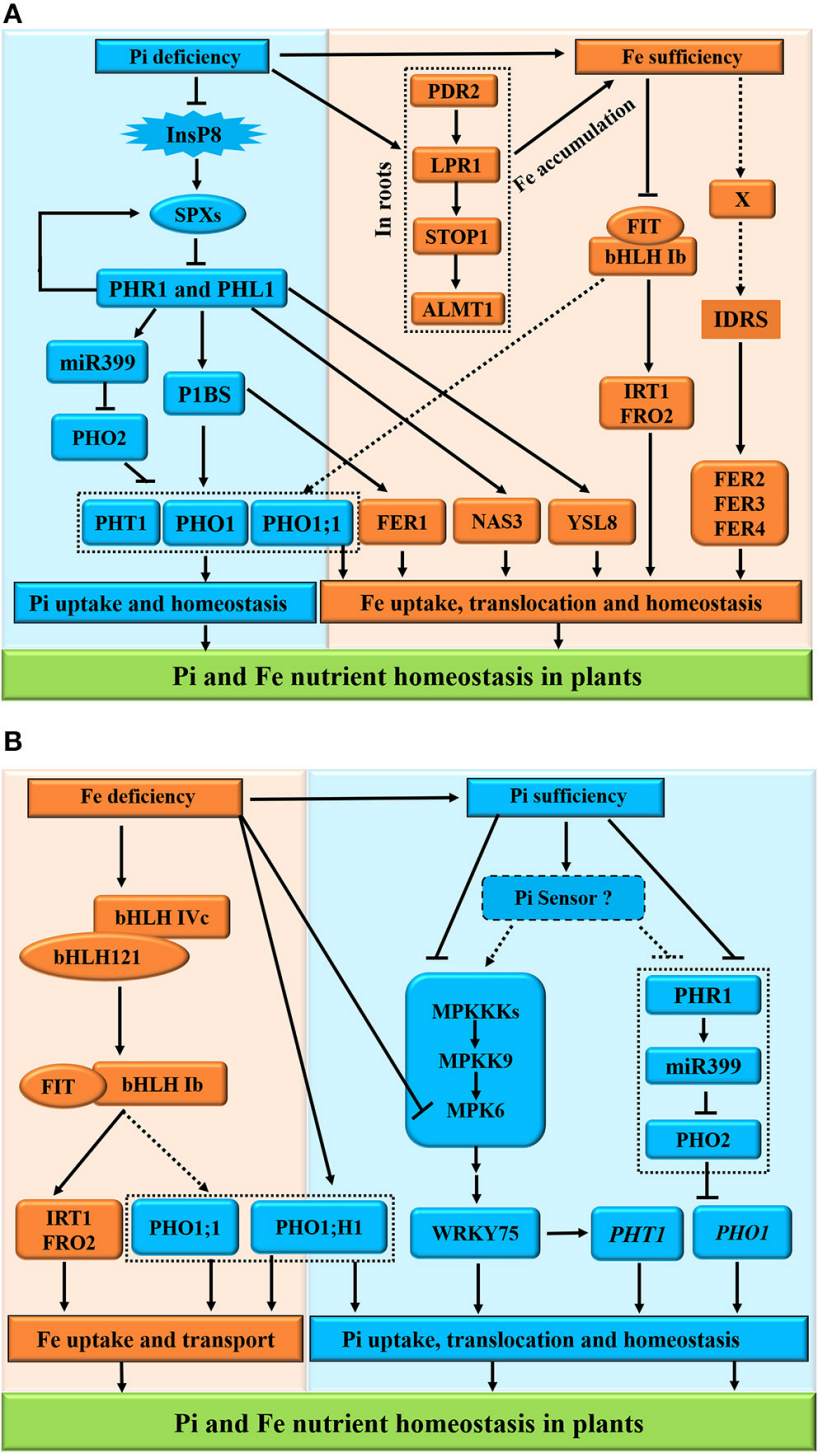
Schematic representation of Pi and Fe homeostasis interactions in plants. The phosphate (Pi) deficiency promotes the iron (Fe) accumulation **(A)**; conversely, Fe deficiency induces the high Pi in plants at the physiological level **(B)**. For the molecular bases of the Pi-Fe cross-talk, the PHR1, PHL1, PHO1;1, PHO1; H1, FER1, STOP1, and MPK6 act as potential integrators of Pi and Fe nutrient signals in plants. **(A)** Under Pi deficiency, the PHR1 and PHL1 function as the core transcription factors controlling the expression of Pi transporters PHT1 and PHO1 *via* the PHR1-miR399-PHO2-signaling pathway. Moreover, PHR1 and PHL1 act as the positive regulators of the transcription of Fe transporters FER1, NAS3, and YLS8 responsible for Fe homeostasis in plants. On the other hand, Pi deficiency also induces the STOP1-modulated signaling to promote Fe accumulation in roots. However, under Fe sufficiency, as a key *cis*-acting element of plant iron homeostasis, IDRS (iron-dependent regulatory sequence, which is bound by unknown factor X) is responsible for the upregulation of ferritin genes *FER2, FER3*, and *FER4*, whereas the central regulator FIT is inactivated to inhibit the expression of *FRO2* and *IRT1*. **(B)** By contrast, Fe deficiency activates the interacting bHLH IVc (bHLH34/104/105/115) and bHLH121 complex to positively regulate the expression of bHLH Ib (bHLH38/39/100/101) and FIT. The bHLH Ib and FIT interact with each other to induce the expression of Fe uptake-related genes *FRO2* and *IRT1* as well as PHO1 Pi transporter genes *PHO1; H1*, and *PHO1;1* for Pi homeostasis. However, the dynamics in Pi also elicits the MAPK-mediated Pi sensing, signaling, and responses in plants, while Fe deficiency reduces the MAPK6 activity to repress the PHT1 family genes downstream of the WRKY75 transcription factor, indicating the role of MPK6 in the coordination of the responses to Pi and Fe nutrients in Pi uptake and homeostasis. The arrows and flat-ended lines indicate the positive and negative influences, respectively. The confirmed influences are presented with solid lines, whereas non-confirmed influences are shown with dashed lines.

Here, we presented the molecular mechanisms of Pi-Fe interactions and proposed models of the interaction between Pi and Fe in plants (Figure 4). Under Pi deficiency, the PHR1 and PHL1 function as the core transcription factors controlling the expression of Pi transporters PHT1 and PHO1 *via* the PHR1-miR399-PHO2-signaling pathway. Moreover, PHR1 and PHL1 act as the positive regulators of the transcription of Fe transporters FER1, NAS3, and YLS8 responsible for Fe homeostasis in plants (Figure 4A). On the other hand, Pi deficiency also induces the STOP1-modulated signaling cascade (PDR2-LPR1-STOP1-ALMT1) (Dong et al., 2017; Godon et al., 2019; Hanikenne et al., 2021) to promote Fe accumulation in roots (Figure 4A, the right panel). However, under Fe sufficiency, as a key *cis*-acting element of plant iron homeostasis, IDRS (iron-dependent regulatory sequence, which is bound by unknown factor X) is responsible for the upregulation of ferritin genes *FER2, FER3*, and *FER4*, whereas the central regulator FIT is inactivated to inhibit the expression of *FRO2* and *IRT1* (Bournier et al., 2013; Chen et al., 2018). By contrast, Fe deficiency activates the interacting bHLH IVc (bHLH34/104/105/115) and bHLH121 complex to positively regulate the expression of bHLH Ib (bHLH38/39/100/101) and FIT (Cai et al., 2021). The bHLH Ib and FIT interact with each other to induce the expression of Fe uptake-related genes *FRO2* and *IRT1* as well as PHO1 Pi transporter genes *PHO1; H1* and *PHO1;1* (Yi and Guerinot, 1996; Vert et al., 2002; Chen et al., 2018) for Pi homeostasis (Figure 4B). However, the dynamics in Pi also elicits the mitogen-activated protein kinase (MPK)-mediated Pi sensing, signaling, and responses in plants (Lei et al., 2014), while Fe deficiency reduces the MPK6 activity to repress the PHT1 family genes downstream of the WRKY75 transcription factor (Devaiah et al., 2007; Takahashi et al., 2007; Figure 4B, the right panel), indicating the role of MPK6 in the coordination of the responses to Pi and Fe nutrients in Pi uptake and homeostasis (López-Bucio et al., 2019).

Taken together, for the molecular bases of the cross-talks between Pi and Fe, the proteins PHR1, PHL1, PHO1;1, PHO1; H1, FER1, STOP1, and MPK6 act as the potential integrators of Pi and Fe nutrient signals in plants.

## Interactions Between Zn and Fe Nutrients in Plants

### Zn Availability Affects Fe Uptake and Homeostasis in Plants

Zinc and iron are essential micronutrients for all living organisms and play a crucial role in plant growth and development (Marschner, 2012). Zinc and iron deficiency reduces plant growth and affects grain yield and quality (Casterline et al., 1997). The antagonistic relationship between Fe and Zn in plants, and between Zn or Fe deficiency and Pi concentration has been examined in plants (Jain et al., 2013; Zargar et al., 2013, 2015; Rai et al., 2015). An increase in Zn concentration resulted in a decrease in the concentration of Fe in the shoots of *A. thaliana*; however, Fe concentration was not affected in the roots of *A. thaliana*. The concentration of Zn in the shoot of plants was positively correlated with the zinc concentration of the growth medium and was not affected by iron concentration (Shanmugam et al., 2011). Although the leaves of *Thlaspi caerulescens* had high-concentration Zn, the root Zn content was low (van de Mortel et al., 2006). A decrease in the Fe content of shoots of *T. caerulescens* was attributed to a high concentration of Zn in the growth medium. Contrarily, increasing the concentration of Fe in the medium resulted in a decrease in the concentration of Zn in the shoots (Pineau et al., 2012). High-Fe concentration reduced the effects of Zn toxicity in *A. thaliana* exposed to high-soil Zn. Therefore, the competition between Zn and Fe plays an important role in high-Zn tolerance in plants (Fukao et al., 2011; Shanmugam et al., 2011; Pineau et al., 2012). Furthermore, recent study has shown that the excess of Zn application mimics Fe-deficiency-induced chlorosis in plant shoots, and analysis of Fe-related morphological, physiological, and regulatory responses in plants subjected to excess Zn reveals that the enhanced Zn uptake closely mimics Fe deficiency, resulting in low-chlorophyll concentrations but high-ferric-chelate reductase activity (Lešková et al., 2017). Lešková et al. (2017) further found that these responses were not resulted from the high-Zn-reduced Fe uptake at the level of transport, whereas Zn simulated the transcriptional response of Fe-regulated key genes. This finding indicates that excess Zn disturbs Fe homeostasis at the level of Fe sensing.

### Fe Availability Affects Zn Uptake and Homeostasis in Plants

Fe deficiency in agricultural soil is a worldwide problem and could lead to a significant reduction in crop yield production (Mori, 1999). Recent studies have reported that Fe deficiency accelerates excess uptake and accumulation of Mn and Zn (Kobayashi et al., 2012). The growth of *Zea mays* under both Fe and Zn deficiency was significantly higher than that under only Fe deficiency (Kanai et al., 2009). Fe deficiency is usually a major inducer of Zn toxicity, while plant symptoms under excess Zn resemble symptoms of Fe-deficient plants. Increasing Fe concentration alleviates the effects of Zn toxicity in *A. thaliana* (Shanmugam et al., 2011), while Fe deficiency inhibits root elongation (Gruber et al., 2013; Satbhai et al., 2017). However, Zn deficiency slightly promotes early root growth (Bouain et al., 2018). On the other hand, when grown in excess Zn medium, the inhibition of *Arabidopsis* growth was sufficiently recovered by Fe addition (Zargar et al., 2015), indicating that the appropriate application of Fe can significantly alleviate the effects of Zn toxicity in plants when grown in Zn-contaminated soil. Moreover, Fe-mediated plant Zn tolerance may be sustained by the fine-tuned Zn homeostasis mechanism in *Arabidopsis* plants, which prevent high Zn uptake through Fe-regulated metal transporters responsible for Zn tolerance (Shanmugam et al., 2011). This suggests a cross-talk between excess Zn and Fe deficiency in plants.

### Molecular Basis of Zn and Fe Interaction in Plants

**Figure 5:**
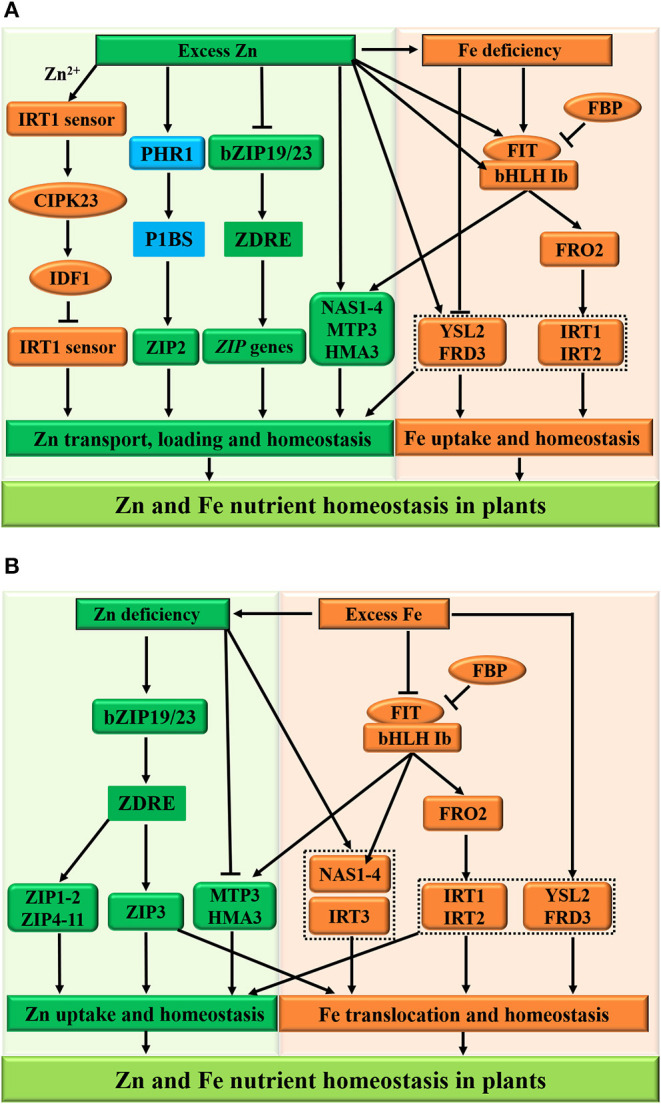
Schematic representation of Zn and Fe homeostasis cross-talk in plants. The excess zinc (Zn) results in the iron (Fe) deficiency **(A)**, whereas excess Fe reduces the Zn concentration in plants at the physiological level **(B)**. For the molecular bases of the Zn-Fe homeostasis cross-talk, the FIT protein, ZIP family transporters (ZIP3 and IRT3), and metal tolerance proteins (FRD3, MTP3, and HMA3) function as potential integrators of Zn and Fe nutrient signals in plants. **(A)** Under excess Zn conditions, the IRT1 acts as a transceptor to sense excess Zn^2+^ and orchestrates its own degradation to protect plants from highly reactive Zn^2+^, while the low-Zn-adaptive bZIP19/23 transcription factors are inactivated and inhibit the plant ZIP gene family transporters (such as ZIP5 and ZIP9) to regulate Zn homeostasis. In addition, metal tolerance proteins MTP3 and HMA3 are activated to facilitate Zn loading into xylem. However, the ZIP2 subfamily genes are independent of the bZIP19/23-mediated pathway and are under the control of the PHR1-dependent pathway, resulting in the Zn overaccumulation in plants. Furthermore, the expression of *MTP3* and *HMA3* is partially dependent on the FIT and contributes to Zn tolerance, while the FIT-independent FRD3 and YSL2 function in Zn and Fe homeostasis, respectively. **(B)** On the other hand, the deficient Zn-regulated ZIP family (such as ZIP3 and IRT3) and NAS1/4 transporters, contributing to Fe uptake and transport, are induced during Zn deficiency, leading to Fe sufficiency in plants. Consequently, the FBP (FIT-binding protein) inhibits the FIT activity, and the bHLH Ib and FIT transcription factors are repressed in adaptive to Zn deficiency and excess Fe, and then expression of *NAS1/4, FRO2*, and *IRT1/2* are downregulated at the transcriptional level to modulate Zn and Fe homeostasis in plants. The arrows and flat-ended lines indicate the positive and negative influences, respectively.

Based on the current literature, the interplay between Zn and Fe homeostasis at the molecular level in plants is discussed here (Figure 5). The FER-LIKE IRON DEFICIENCY-INDUCED TRANSCRIPTION FACTOR (FIT) protein (Colangelo and Guerinot, 2004; Schwarz and Bauer, 2020), ZIP family transporters (ZIP3 and IRT3) (Mäser et al., 2001), and metal tolerance proteins, including FERRIC REDUCTASE DEFECTIVE3 (FRD3), MTP3 (a member of the Cation Diffusion Facilitator family), and HMA3 (belonging to the P1B-type ATPase family) are potential molecular integrators of Zn and Fe signaling in plants (Rogers and Guerinot, 2002; Becher et al., 2003; Arrivault et al., 2006; van de Mortel et al., 2006; Haydon and Cobbett, 2007; Haydon et al., 2012). Under high Zn conditions (Figure 5A), *Arabidopsis* plants maintain Zn homeostasis by inhibiting the activities bZIP19/23 transcription factors and ZIP gene family transporters (ZIP5 and ZIP9) (Assuncao et al., 2010). Additionally, metal tolerance proteins MTP3 and HMA3 are activated to facilitate Zn loading into xylem (Arrivault et al., 2006; Briat et al., 2015). However, the ZIP2 subfamily genes may be independent of the bZIP19/23-mediated pathway and are under the control of the PHR1-dependent pathway due to the existence of the P1BS *cis*-acting element in their promoters (see Figure 3A), resulting in Zn overaccumulation in plants (Assuncao et al., 2010).

Under high Zn conditions (Figure 5A), the activities of the ZIP family transporters, which promote Fe uptake and transport, are repressed, leading to Fe deficiency in plants (Shanmugam et al., 2011; Li et al., 2013). On the other hand, this Zn excess provokes Fe deficiency responses at the regulatory level and affects the transcript levels of Fe-deficiency-responsive genes. Particularly, the transcript levels of all Fe-deficiency-induced bHLH Ib (bHLH38/39/100/101) and FIT transcription factors experience a strong upregulation in plant roots after prolonged exposure to external high-Zn concentrations (Lešková et al., 2017; see Figure 5A). Under Zn and Fe deficiencies, plants maintain homeostasis by activating the FIT transcription factor and by upregulating the expression of FERRIC IRON REDUCTASE 2 (FRO2) and IRON-REGULATED TRANSPORTER1/2 (IRT1/2) to increase the uptake and transport of Zn and Fe (Yuan et al., 2008; Wang et al., 2013). Furthermore, the expression of *MTP3* and *HMA3*, which regulates Zn tolerance, is partially dependent on the activities of FIT, whereas *FRD3* and *YSL2* are involved in Zn and Fe homeostasis, respectively (Schaaf et al., 2005; Scheepers et al., 2020; Zang et al., 2020). A more recent report has uncovered that the plant IRT1 acts as a metal transporter receptor (also called transceptor or sensor) to directly sense the soil excess of its non-iron metal substrate, such as Zn^2+^, and to orchestrate its own vacuolar degradation in the cytoplasm (Dubeaux et al., 2018; see Figure 5A, the left panel). In brief, when Zn is present in excess, the plasma membrane-localized IRT1 as a transceptor senses the excess of Zn^2+^ in the cytoplasm and binds Zn^2+^ to its histidine-rich region. Subsequently, the IRT1 sensor can recruit the calcium-dependent CBL-INTERACTING PROTEIN KINASE 23 (CIPK23), which phosphorylates the variable region of IRT1. The phosphorylated IRT1 is ubiquitinated by the E3 ubiquitin ligase IRT1-DEGRADATION FACTOR1 (IDF1) and degraded in the vacuole. This new finding also provides insights into the defined molecular mechanism by which the plant Fe transceptor transports and senses the elevated Zn^2+^ concentrations and integrates Zn^2+^-dependent regulations to maximize Fe uptake and protect plants from toxic Zn.

Under low-Zn conditions (Figure 5B), the low-Zn-adaptive bZIP19/23 transcription factors are activated and induce the expression of plant ZIP gene family transporters (such as ZIP1-11) to regulate Zn uptake and homeostasis. However, the metal tolerance proteins MTP3 and HMA3 are inactivated to inhibit Zn loading into xylem (Morel et al., 2009), whereas the upregulated expression of *ZIP3* is dependent on the bZIP19/23 transcription factors and contributes to Fe translocation (Hanikenne et al., 2021), resulting in a high level of Fe concentrations in plants (Figure 5B); consequently, the FIT-independent FRD3 (Pineau et al., 2012; Scheepers et al., 2020) and YSL2 (Schaaf et al., 2005; Zang et al., 2020) function in Zn and Fe homeostasis, respectively. In addition, the IRT3 and NAS1/4 transporters, contributing to Fe uptake and transport, are also induced during Zn deficiency (Talke et al., 2006), leading to Fe sufficiency in plants. Consequently, the FBP (FIT-binding protein) inhibits the FIT activity (Chen et al., 2018), and the bHLH Ib and FIT transcription factors are repressed in adaptive to Zn deficiency and excess Fe, and then expression of *NAS1/4, FRO2*, and *IRT1/2* is downregulated at the transcriptional level to modulate Zn and Fe homeostasis in plants.

## The Common Signals Involved in Pi, Zn, And Fe Nutrient Homeostasis in Plants

Mineral nutrients availability affects the gene regulatory networks, which, in turn, initiate the expression of genes-regulating ion homeostasis in plants, resulting in the regulation of plant growth and development (Bouain et al., 2019). To understand the tripartite cross-talk between Pi, Zn, and Fe in plants, it is necessary to identify the molecular actors that integrate Pi, Zn, and/or Fe signaling and homeostasis.

Recently, some studies have revealed that the MYB transcription factor, such as PHR1, regulated the genes essential for Pi, Zn, and Fe uptake and homeostasis in plants (Rouached et al., 2011; Bournier et al., 2013; Khan et al., 2014; Briat et al., 2015; Kumar et al., 2021). Therefore, PHR1 acts as the first common regulator of Pi, Zn, and Fe homeostasis and serves as an integrator of multiple mineral signals in plants (Briat et al., 2015; see Figures 3, 4). In this review, the role of PHR1 in the cross-talks between two minerals has been examined in detail, including Pi and Zn (see Figure 3), and Pi and Fe (see Figure 4). PHR1 functions as a core transcription factor in the expression of the Pi transporter gene (PHT1 and PHO1; H1). Moreover, PHR1 also regulates the expression of *miR399* and *PHO2*. In response to Pi deficiency during Pi-Zn cross-talk, PHR1 or its ortholog OsPHR2 (in rice) may act as a positive regulator of the ZIP family transporters for Zn accumulation in plant shoots regarding the presence of PIBS *cis*-element in plant *ZIP* genes (see Figure 3A) and the induction of these Zn transporter genes in response to Pi deficiency (Ding et al., 2021; Kumar et al., 2021). Furthermore, the transcription of Fe homeostasis-associated genes is dependent on the PHR1 activity, including the activity of *FER1* (Bournier et al., 2013; Briat et al., 2015; also see Figure 4). The regulatory roles of these PHR1-dependent genes in the control of Pi, Zn, and/or Fe uptake have been partially studied in different plant species. PHT1;1, PHO1, and PHO1; H3 are coordinatively involved in the cross-talk between Pi and Zn homeostasis in *Arabidopsis* (Bouain et al., 2014b; Khan et al., 2014; Kisko et al., 2015). In the case of Pi-Fe interaction, the expression of *FER1* gene-encoding ferritin is upregulated under Pi deficiency conditions in a PHR1-dependent manner (Petit et al., 2001; Bournier et al., 2013). Additionally, there was an increase in the expression of *NAS3* and *YSL8*, which are responsible for Fe transport during Pi deficiency (Thibaud et al., 2010). Furthermore, *phr1* × *phl1* double mutation in *Arabidopsis* affected the expression of the Fe homeostasis-associated gene (Bournier et al., 2013; Briat et al., 2015), suggesting that besides PHR1, PHL1 may serve as an integrator in the cross-talk between Pi deficiency and Fe stress-signaling pathways in plants.

The bZIP23 transcription factor has been identified as a molecular integrator in the cross-talk between Pi and Zn homeostasis. Kisko et al. (2018) reported that the *Lyso-PhosphatidylCholine AcylTransferase 1* (*LPCAT1*) gene was downregulated by the bZIP23 TF and serves as the key determinant of shoot Pi accumulation under Zn deficiency. Additionally, other key molecular players have been identified in plants in response to Pi and Fe deficiencies. Dai et al. (2016) demonstrated that *OsWRKY74*, a WRKY transcription factor in rice, regulates phosphate deficiency tolerance, and was involved in Fe deficiency responses. There was an increase in the expression of *OsWRKY74* in rice under Pi deficiency, resulting in higher Fe accumulation compared with that in the wild type. This result indicates that OsWRKY74 is involved in the cross-talk between Pi and Fe deficiency signaling. Moreover, LPR1 (LOW PHOSPHATE ROOT1), PDR2 (PHOSPHATE DEFICIENCY RESPONSE2), STOP1 (SENSITIVE TO PROTON RHIZOTOX-ICITY1), and ALMT1 (ALUMINUM-ACTIVATED MALATE TRANSPORTER1) participate in the interaction of Pi and Fe to promote Fe accumulation in roots in response to Pi deficiency (Ticconi et al., 2004; Svistoonoff et al., 2007; Balzergue et al., 2017; Mora-Macías et al., 2017). Furthermore, studies have shown that MPK6 integrates Pi and Fe responses and coordinates the homeostasis between Pi deficiency and Fe signaling in *Arabidopsis* (López-Bucio et al., 2019). Zargar et al. (2013) examined the response to high Zn and Fe deficiency in *Arabidopsis*.

The bHLH Ib (bHLH38/39/100/101) and FIT transcription factors are regulated in response to high Zn application (Lešková et al., 2017), indicating that these Fe-deficient regulatory proteins may act as important integrators in the interaction between Fe deficiency and Zn excess. Moreover, under both Fe-deficient and high-Zn conditions, several metal ion transporters, including NRAMP4, OPT3, and MRP3, and membrane protein, including SYP122, could act as molecular integrators in the regulation of the homeostatic cross-talk that occurs in response to high Zn and Fe deficiency. There is a need for detailed studies on the gene regulatory networks involved in the Zn-Fe cross-talk in plants.

It is worth noting that the signaling intermediates, such as calcium ion (Ca^2+^) and reactive oxygen species (ROS), play critical roles in the plant iron and zinc nutrient homeostasis (Gratz et al., 2019; von der Mark et al., 2021). Very recent studies have revealed the molecular machineries of how Ca^2+^ signaling is involved in the regulation of FIT and IRT1 activities and is responsible for plant iron acquisition and homeostasis (Gratz et al., 2019; Khan et al., 2019). Gratz et al. (2019) also found that the CBL-INTERACTING PROTEIN KINASE (CIPK11) expression and cytosolic Ca^2+^ concentrations are induced during Fe deficiency and conformed CIPK11 as a FIT interactor. They further proposed that the second message Ca^2+^-triggered CBL1/9 mediated the CIPK11 activation, and subsequent CIPK11-dependent phosphorylation-modulated FIT activity shifted inactive into active FIT to enhance plant iron uptake in response to calcium signaling. Conversely, calcium-promoted direct interaction between the C2-domain containing peripheral membrane protein-ENHANCED BENDING1 (EHB1, belonging to the CAR family member) and metal transporter IRT1 inhibited plant iron acquisition required for the prevention of metal toxicity (Khan et al., 2019). These novel findings uncover that these biochemical links between Fe/Zn availability and the second message Ca^2+^ signal-decoding machinery represent a defined environment-sensing mechanism to adjust nutrient uptake and homeostasis.

Reactive oxygen species (ROS) as an intermediate signal play a central role in the regulation of plant stress responses (Czarnocka and Karpiński, 2018; Smirnoff and Arnaud, 2019). The prolonged Fe deficiency-induced hydrogen peroxide (H_2_O_2_) elicits signaling events, leading to the inhibition of plant Fe uptake *via* the FIT inactivation. To test this, the very recent study has shown that the increased H_2_O_2_ in plants negatively affects the gene expression of downstream transcription factors FIT, bHLH Ib (bHLH038/039/100/101) and POPEYE (PYE), and subsequently inhibits the Fe uptake-related FIT target genes *IRT1* and *FRO2* by analysis of Fe homeostasis transcription cascade (von der Mark et al., 2021). This finding reveals that ROS coordinates the transcriptional responses to Fe availability in plants. On the other hand, excess Fe increases ferritin abundance to store Fe and protect plants from Fe-induced oxidative stress, whereas Fe excess inhibits root growth. It is well-demonstrated that ROS signaling participates in the Fe homeostasis and ferritin accumulation in plants (Ravet et al., 2009; Briat et al., 2010). Reyt et al. (2015) reported that ROS homeostasis mediated the interaction between ferritin function and root system architecture (RSA) remodeling in response to Fe excess. Moreover, the ROS-activated SMR5/7 cyclin-dependent kinase inhibitors controlled the root meristem cell cycle arrest in the interplay between Fe and RSA. These two cases mentioned above represent the mechanisms for ROS signaling to coordinate Fe uptake and redistribution in plant responses to Fe stress.

## Conclusion and Perspectives

In terrestrial ecosystems, higher plants are sessile organisms and faced with variable environmental stresses, including soil nutrient deficiency and highly reactive metals, which significantly affect plant survival and development. In particular, crop plants grown in soils are exposed to nutrient stresses during their lifecycle, such as low or high amount of essential mineral elements, including N, P, S, Zn, and Fe. Therefore, plants have evolved effective mechanisms to coregulate these stimuli to maintain nutrient homeostasis. To date, the unexpected cross-talks between macro- and micro-mineral elements in plants have long been recognized at morphological and physiological levels. Nevertheless, despite their fundamental importance, the molecular bases, regulatory networks, and biological significance of these interactions in plants remain poorly understood. Recently, several pieces of research have emphasized the analysis of plant nutrition, considering two or more nutrient combinations, and proposed that N, Pi, Zn, and/or Fe homeostasis are highly regulated in plants at different hierarchic levels (Lešková et al., 2017; Dubeaux et al., 2018; Bouain et al., 2019; Brumbarova and Ivanov, 2019). On one hand, the synergistic effects between N and P, and N and Zn in rice plants have been demonstrated at the nutrient signaling and transport levels (Medici et al., 2019; Ji et al., in press). On the other hand, the antagonistic interactions between P and Zn, P and Fe, and Zn and Fe have been noted in the area of plant nutrition at the transcriptional response, nutrient sensing, signaling, and transport levels (Zheng et al., 2009; Khan et al., 2014; Lešková et al., 2017; Dubeaux et al., 2018; Gratz et al., 2019; Ding et al., 2021; von der Mark et al., 2021). Moreover, in these excellent studies, some important molecular integrators, including nutrient sensors (NRT1.1 and IRT1), central regulators (PHR1, bZIP19/23, bHLH Ib and FIT), nutrient transporters/enzymes (PHT1, PHO1;1, ZIPs, FER1, LPCAT1, and MAPK6), and chemical/biochemical signals (Ca^2+^, ROS, and CIPK11/23), have been identified and characterized in plants during these complex processes. Future studies will need to undertake integrative studies in order to reveal the underlying mechanisms by which plants (including mycorrhizal plants) coordinate these multiple nutrient stresses signaling at the whole-plant systemic level through the combination of gene coexpression analysis, multiple “omics” approaches, and gene editing. This will help us further understand the biological significance of these cross-talks and will help the scientists and farmers develop new strategies for field nutrient management. For example, by this way, the combined application of N and Zn fertilizers in the field is an effective method for enhancing both Zn nutrition and N utilization in crop plants, which is beneficial for humans with diets, while the appropriate supply of P or Fe fertilizer is a master strategy to alleviate crop uptake of excessive Zn when grown in high-Zn soil (Ding et al., 2021; Ji et al., in press).

In addition, land plants largely depend upon their associated microbes for growth promotion and nutrient availability under various stresses (Edwards et al., 2015; Andreote and Silva, 2017; Kaul et al., 2021). If we better study the beneficial interactions between plants and their associated microbiota in the rhizosphere, such as plant growth-promoting rhizobacteria (PGPR) (Kumar and Verma, 2018), arbuscular mycorrhizal (AM) fungi (Genre et al., 2020), and endophytic fungi (Tang et al., 2019), we can properly manipulate the PGPR and AM fungi in soils for facilitating plant nutrient acquisition (White, 2019) and alleviating plant responses to toxic metal stresses (Rajkumar et al., 2012; Tak et al., 2013), and can lead the modern agriculture to a master exploration of these universal nutrient sources, improving crop yield and agricultural sustainability.

In conclusion, we emphasize again the systemic development of the integrative study of cross-talk between macro- and micro-mineral elements uptake and signaling in land plants will be of great significance and essential for sustainable agricultural and forestry development all around the world.

## Author Contributions

XX and MT conceived and designed this study. XF and XX wrote the manuscript. HC and XZ proposed related theories and assisted with the interpretation of some references. All the authors have read, edited, and approved the current version of the manuscript.

## Funding

This study was supported by grants from the Natural Science Foundation of Guangdong Province in China (Grant No. 2018A030313141), the Key Projects of Guangzhou Science and Technology Plan (Grant No. 201904020022), the National Natural Science Foundation of China (Grant No. 31800092, 32071639), and the High-Level Talent Start Funding Project of South China Agricultural University (Grant No. 221144).

## Conflict of Interest

The authors declare that the research was conducted in the absence of any commercial or financial relationships that could be construed as a potential conflict of interest.

## Publisher's Note

All claims expressed in this article are solely those of the authors and do not necessarily represent those of their affiliated organizations, or those of the publisher, the editors and the reviewers. Any product that may be evaluated in this article, or claim that may be made by its manufacturer, is not guaranteed or endorsed by the publisher.
